# Iron Oxide Nanorings and Nanotubes for Magnetic Hyperthermia: The Problem of Intraparticle Interactions

**DOI:** 10.3390/nano11061380

**Published:** 2021-05-24

**Authors:** Raja Das, Javier Alonso Masa, Vijaysankar Kalappattil, Zohreh Nemati, Irati Rodrigo, Eneko Garaio, José Ángel García, Manh-Huong Phan, Hariharan Srikanth

**Affiliations:** 1 Faculty of Materials Science and Engineering, Phenikaa University, Hanoi 12116, Vietnam; 2 Phenikaa Research and Technology Institute (PRATI), A&A Green Phoenix Group, 167 Hoang Ngan, Hanoi 13313, Vietnam; 3 Departamento CITIMAC, Universidad de Cantabria, 39005 Santander, Spain; alonsomasaj@unican.es; 4 Department of Physics, University of South Florida (USF), Tampa, FL 33620, USA; vijaysankar@mail.usf.edu (V.K.); zohre.nematy@gmail.com (Z.N.); phanm@usf.edu (M.-H.P.); 5 Departamento de Electricidad y Electrónica, Universidad del País Vasco (UPV/EHU), 48940 Leioa, Spain; irati.rodrigo@bcmaterials.net; 6 Departamento de Física Aplicada, Universidad Pública de Navarra (UPN), 31006 Pamplona, Spain; eneko.garayo@unavarra.es; 7 Departamento de Física, Universidad del País Vasco (UPV/EHU), 48940 Leioa, Spain; joseangel.garcia@ehu.eus

**Keywords:** magnetic nanoparticles, biomedical applications, nanomagnetism, magnetic interaction, magnetic hyperthermia

## Abstract

Magnetic interactions can play an important role in the heating efficiency of magnetic nanoparticles. Although most of the time interparticle magnetic interactions are a dominant source, in specific cases such as multigranular nanostructures intraparticle interactions are also relevant and their effect is significant. In this work, we have prepared two different multigranular magnetic nanostructures of iron oxide, nanorings (NRs) and nanotubes (NTs), with a similar thickness but different lengths (55 nm for NRs and 470 nm for NTs). In this way, we find that the NTs present stronger intraparticle interactions than the NRs. Magnetometry and transverse susceptibility measurements show that the NTs possess a higher effective anisotropy and saturation magnetization. Despite this, the AC hysteresis loops obtained for the NRs (0–400 Oe, 300 kHz) are more squared, therefore giving rise to a higher heating efficiency (maximum specific absorption rate, SAR_max_ = 110 W/g for the NRs and 80 W/g for the NTs at 400 Oe and 300 kHz). These results indicate that the weaker intraparticle interactions in the case of the NRs are in favor of magnetic hyperthermia in comparison with the NTs.

## 1. Introduction

Cancer treatment through magnetic hyperthermia relies on delivering magnetic nanoparticles (MNPs) to a tumor area in order to deactivate cancer cells by locally raising the temperature while avoiding collateral damage to healthy tissues [1,2,3,4]. Since it was proposed by Gilchrist et al. in 1957 [5], the technique has progressively advanced and nowadays there are already a few clinical trials being carried out in different hospitals around the world [3,6]. In addition, in the last years, different strategies have been devised to improve the theranostic efficiency of these nano-agents such as combining magnetic hyperthermia and photothermia in magneto-plasmonic nanostructures or using magneto-mechanical actuation for further disrupting the cancer cells [7,8,9,10,11,12,13,14,15]. Despite a large number of advancements in the field, there are still several issues that warrant further studies such as improving the delivery efficiency of the MNPs to the tumor or optimizing the heating efficiency (i.e., the specific absorption rate, SAR) of the MNPs.

Concerning this last issue, one of the most complex problems that arises in the effort of increasing the SAR is the problem of magnetic interactions [16,17]. Interparticle magnetic interactions appear when the separation between the individual MNPs is small enough. These types of interactions tends to give rise to an undesired agglomeration of the MNPs inside the human body, which can be challenging [18,19]. The presence of these interactions has been reported to reduce the heating efficiency of the MNPs. The degradation of the SAR with increasing interparticle interactions can be, in principle, associated with the magnetic disorder and additional anisotropies introduced by magnetic interactions [20,21,22,23,24,25]. The hysteresis losses are often reduced in the agglomerated MNPs during the application of the AC field, resulting in lower SAR values in comparison with the case of the well-separated MNPs [26]. There are, nevertheless, exceptions to this rule: for example, when the interparticle magnetic interactions can be controlled so that they give rise to specific arrangements of the MNPs such as chains, it has been demonstrated that their heating efficiency can increase in comparison with the non-interacting MNPs [27,28]. However, it is not straightforward to obtain MNPs forming such arrangements.

Apart from interparticle magnetic interactions, one must also consider the presence of intraparticle magnetic interactions as has been reported in several experimental and theoretical works [29,30,31,32,33]. For example, when the MNPs are multigranular or multidomain, short-range dipolar interactions between nanograins forming part of a nanostructure can also impact their magnetic response and therefore their heating efficiency [34]. To this respect, in the last years several new magnetic nanostructures for magnetic hyperthermia have been brought forward as an alternative to the typically employed spherical single domain MNPs. Among these, we have synthesized magnetic nanodiscs, nanoflowers and core/shell MNPs, for example [35,36,37,38]. By changing the morphology of these nanostructures, we can improve their heating efficiency while exploiting new biomedical capabilities. Recent SANS experiments in multigranular nanoflowers with strong interactions between the nanograins have revealed that a comparatively large heating efficiency can be obtained in these nanostructures due to their intrinsic enhanced spin disorder [38]. Other good examples of these kinds of novel MNPs are iron oxide nanotubes and nanorings [39,40]. These nanostructures have recently been proposed as promising candidates for combined drug delivery and magnetic hyperthermia therapy: by making the particles hollow, the surface area available for attaching drug molecules to the MNPs is increased [41,42]. In addition, the tunable aspect ratio and morphology of these nanostructures provide us with an additional degree of control over their magnetic response.

To better understand the effect of intraparticle interactions in the magnetic hyperthermia response of multigranular nanostructures, in this work we focused on the study of Fe_3_O_4_ nanorings (NRs) and nanotubes (NTs). Both nanostructures present a similar wall thickness but very different lengths (55 nm for NRs and 470 nm for NTs) and different types of nanograins (elongated rods for NTs and spherical nanograins for NRs). In this way, the intraparticle interactions were modified. On the other hand, for all of the measurements, the concentration of the nanostructures was kept the same for both samples in order to “homogenize” the effect of the interparticle interactions. By using transmission electron microscopy (TEM) and X-ray diffraction (XRD), we determined the composition, morphology and size of both nanostructures. In addition, magnetic measurements, zero-field-cooling/field-cooling (ZFC/FC) curves and hysteresis loops (M-H curves) provided us with a depiction of the magnetic behavior of NRs and NTs as a function of temperature (10–300 K) and magnetic field (0–5 T). These results were complemented with transverse susceptibility (TS) measurements, which revealed the differences in the effective anisotropy of both samples. Finally, AC hyperthermia measurements were carried out using a homemade setup and the SAR vs. field curves (*H* = 0–400 Oe, *f* = 300 kHz) were compared and related to the different characteristics of both samples; in particular, to the role of intraparticle interactions.

## 2. Materials and Methods

### 2.1. Synthesis of Fe_3_O_4_ Nanotubes and Nanorings

Fe_3_O_4_ NTs and NRs were synthesized using a two-step process. First, α-Fe_2_O_3_ NTs and NRs were synthesized using a hydrothermal reaction of FeCl_3_ with NaH_2_PO_4_ and Na_2_SO_4_. In a typical reaction, FeCl_3_·6H_2_O (0.27 g) and Na_2_SO_4_ (0.0195 g) and NaH_2_PO_4_·2H_2_O 0.007 g and 0.014 g for α-Fe_2_O_3_ NTs and NRs, respectively, were dissolved in 35 mL of water. The solution was then transferred into a Teflon-lined stainless steel autoclave and heated to 220 °C for 8 h. The α-Fe_2_O_3_ NTs and NRs were then reduced in the presence of hydrogen/argon (7% hydrogen) at 300 °C for 5 h to form Fe_3_O_4_ NTs and NRs.

### 2.2. Structural Characterization

The crystal structure of the nanoparticles was characterized using a Bruker AXS D8 X-ray diffractometer (XRD) working in Bragg Brentano geometry at a Cu K_α_ wavelength. The morphology of the nanoparticles was analyzed using an FEI Morgagni 268 transmission electron microscope (TEM) operating at 60 kV.

### 2.3. Magnetic Characterization

The magnetic measurements were performed in a commercial physical property measurement system (PPMS) from Quantum Design with a vibrating sample magnetometer (VSM) option. All of the magnetic measurements were carried out with the samples in a powder form pressed together inside a gel capsule. The M-T curves were recorded between 5 and 350 K following the zero-field-cooling/field-cooling (ZFC/FC) protocol with an applied field of 50 Oe. On the other hand, M-H loops were measured at 300 K applying fields up to 5 T.

### 2.4. Magnetic Hyperthermia

The AC magnetometry experiments were carried out using a homemade setup [43] on suspensions of 1 mg/mL of NRs and NTs in water. The magnetic field amplitude was varied between 0 and 400 Oe while the frequency was fixed at 300 kHz. Afterwards, the specific absorption rate (SAR), that is, the heating power of the samples under an AC magnetic field, was obtained from the area of dynamic M vs. H hysteresis loops and the fixed frequency. The results were normalized by the mass of the magnetic material according to the saturation magnetization of the samples.

## 3. Results and Discussions

**Figure 1:**
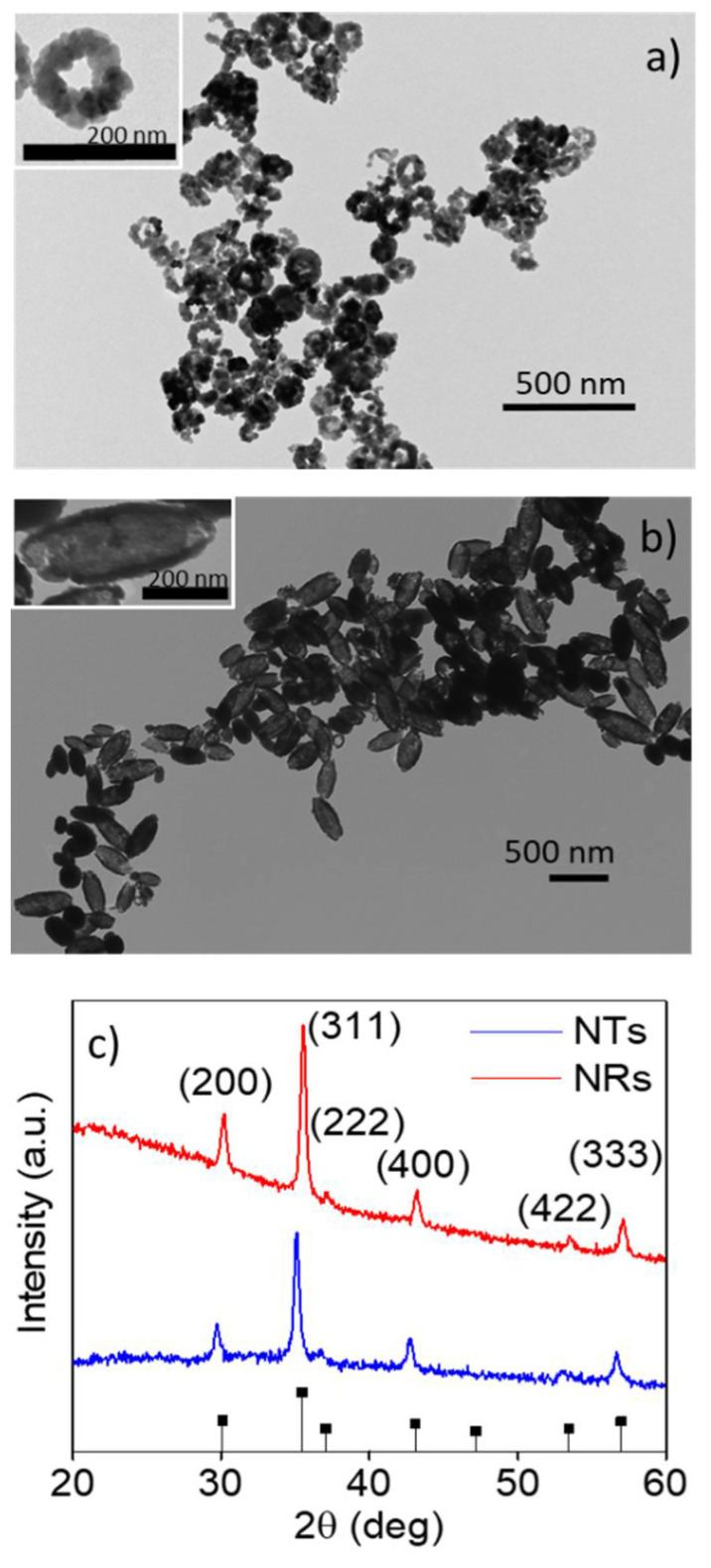
TEM images of (**a**) the iron oxide NRs and (**b**) NTs. In the insets, zoom-in images of a single NR and NT are included. (**c**) XRD data of the iron oxide NRs and NTs. The lower patterns with black squares correspond with the JCPDS data for bulk Fe3O4 (JCPDS No. 19-0629).

The morphology, composition and nanostructure of the NRs and NTs were investigated by a combination of TEM and XRD analyses. In Figure 1a,b, the representative TEM images are included for both samples. Considering the NRs and NTs as hollow cylinders, we determined the corresponding average dimensions from the TEM images as summarized in Table 1: the length, inner and outer diameters and wall thickness (see Figure S1 in the Supplementary Information for the corresponding size distributions). As indicated, both samples presented a similar wall thickness but the radius and especially the length of the NTs was much higher than the NRs. Therefore, the volume of the NTs was greater than that of the NRs. In the inset to these images, we obtained a clearer depiction of the internal nanostructure of these samples. As can be seen, the NRs seemed to consist of semispherical nanograins of around 17 nm while the NTs were composed of nanorod-like units, 70 ± 20 nm long and 7 ± 3 nm wide, bound together as we already described in [39]. The differences in the internal nanostructure of both samples can be related to the differences in the growth process: the ratio of NaH_2_PO_4_·2H_2_O and Na_2_SO_4_ to FeCl_3_·6H_2_O determined the morphology of the nanostructure. In our case, we fixed the concentration of FeCl_3_·6H_2_O and Na_2_SO_4_ and varied the concentration of NaH_2_PO_4_·2H_2_O. We found that the lower concentration of NaH_2_PO_4_·2H_2_O promoted the growth of the NTs and the higher concentration of NaH_2_PO_4_·2H_2_O promoted the growth of the NRs.

On the other hand, XRD patterns (Figure 1c) with well-defined peaks clearly revealed the crystalline nature of both samples. The position of these peaks indicated that the NRs and NTs, after the reduction process in the presence of hydrogen/argon (7% hydrogen), were mainly composed of Fe_3_O_4_ although a small amount of γ-Fe_2_O_3_ could not be discarded. The average crystallite size of Fe_3_O_4_, as calculated using the Debye–Scherrer formula, was around 17 nm for the NRs, supporting the values obtained in the TEM analysis.

**Figure 2:**
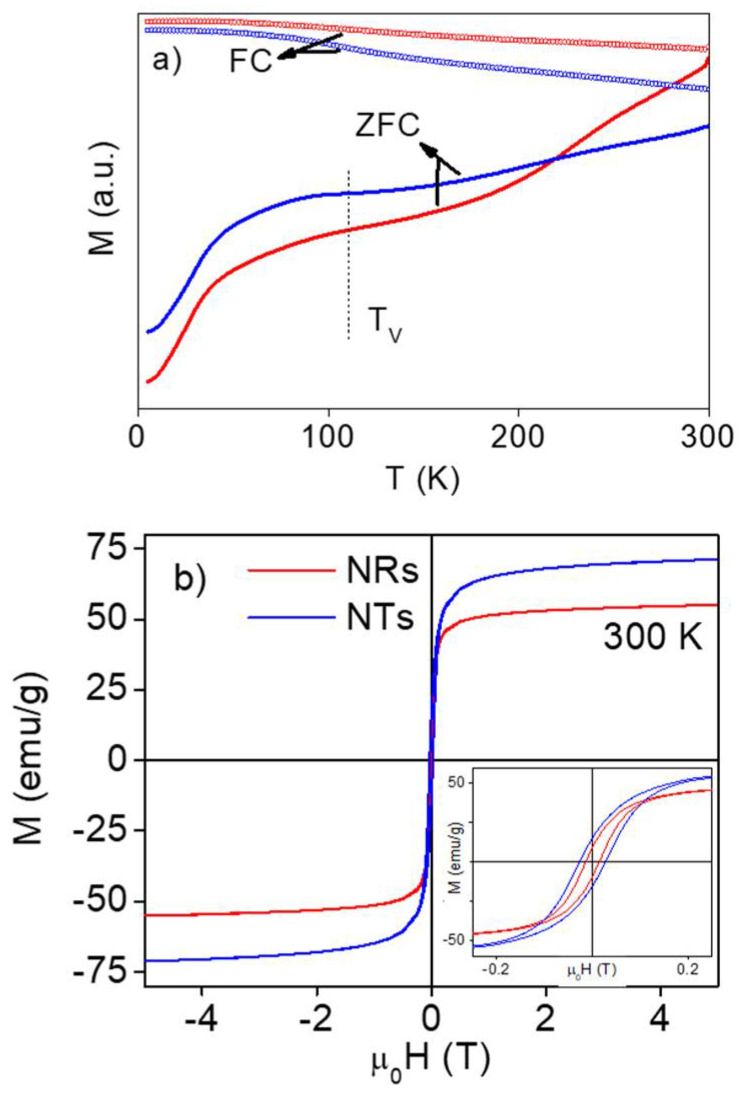
Magnetization measurements for the iron oxide NRs and NTs: (**a**) the ZFC/FC M-T curves measured at 50 Oe (the expected Verwey temperature is indicated by T_V_); (**b**) the M-H loops measured at 300 K with a zoom into the low field region (inset).

**Figure 3:**
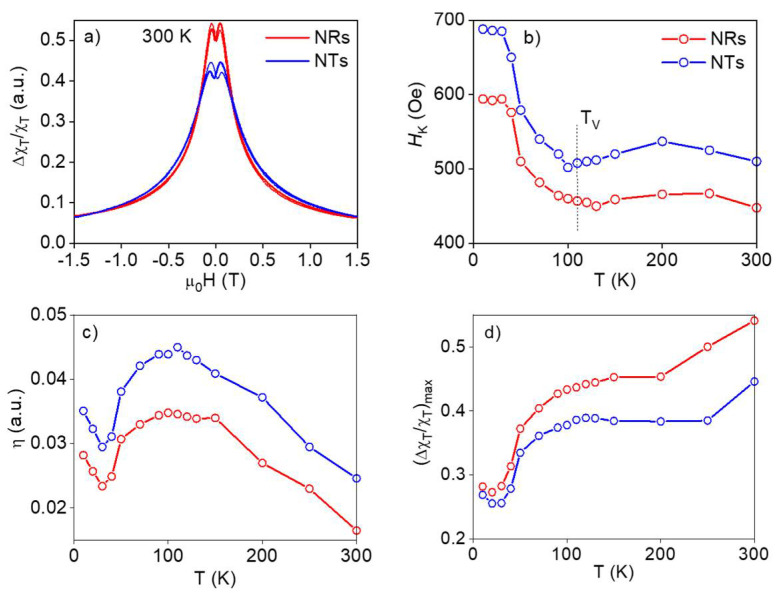
Transverse susceptibility (TS) measurements for the iron oxide NRs and NTs: (**a**) TS bipolar curves measured at 300 K, (**b**) temperature dependence of anisotropy field, H_K_, (the expected Verwey temperature is indicated by T_V_), (**c**) peak height difference (ɳ) and (**d**) maximum change in TS (Δχ_T_/χ_T_)_max_ vs. temperature curves as obtained from the peaks in the TS bipolar curves.

To evaluate how the different nanostructures of the NRs and NTs affected their magnetic response, we first carried out a magnetic characterization both as a function of the magnetic field and temperature. In Figure 2a, we present the ZFC/FC M-T curves measured at 50 Oe between 5 and 300 K. As observed, the ZFC and FC M-T curves for the NRs and NTs followed a similar trend, presenting a high irreversibility and no sign of blocking in the range of temperatures studied. This indicated that, despite the relatively small volume of nanograins forming both samples, neither the NRs nor the NTs were in a superparamagnetic (SPM) state even at room temperature. Although we could not discard that a few of these nanograins were above the blocking temperature (supposing a wide size distribution), the high blocking temperature could be more likely related to an enhancement of the effective anisotropy of the NRs and NTs in comparison with the isolated nanograins. There are several factors that can increase the effective anisotropy including surface disorder, shape effects and, of course, magnetic interactions. Similar ZFC/FC M-T curves have been reported in the literature for interacting iron oxide based MNPs [44,45]. In this respect, it was also remarkable that the Verwey transition (the expected position of the Verwey temperature is indicated in the Figure by T_V_) [46,47,48], which is related to the crystal structure of Fe_3_O_4_ and typically appears as a sudden drop in the magnetization around 110–120 K, was not well-defined in these ZFC/FC M-T curves. A similar smearing of the Verwey transition in other magnetite based MNPs has been associated with the effect of non-stoichiometry, surface disorder and dipolar interactions [48,49,50,51]. We further studied the magnetic responses of the NRs and NTs by measuring the magnetization as a function of the field, the so-called M-H loops, at 300 K (Figure 3b). In this case, we observed several clear differences between the NRs and NTs. The saturation magnetization (M_S_) value for the NTs was around 70 emu/g while for the NRs it was lower, around 55 emu/g. Both M_S_ values were smaller than the theoretical value expected for bulk magnetite (~92 emu/g). There could have been a presence of γ-F_e2_O_3_ in these samples, which would reduce their M_S_ value but as both NRs and NTs were reduced under the same conditions during the synthesis procedure, this would not explain the > 25% difference in the M_S_ of both samples. Therefore, this decrease in M_S_ was probably associated with size effects and a surface disorder, suggesting that these effects were more significant in the case of NRs.

If we focused on the low field region of the magnetic hysteresis (M-H) loops, as depicted in the inset to Figure 2b, we observed that both NRs and NTs presented a clear hysteresis, supporting the absence of an SPM state at room temperature. Despite the relatively small volume of the magnetic nanograins composing both samples, the absence of SPM behavior indicated that the magnetic interactions played an important role. The obtained values for the coercive field and normalized remanence were H_C_ = 140 Oe and M_r_/M_S_ = 0.17 for the NRs while for the NTs, H_C_ = 270 Oe and M_r_/M_S_ = 0.22. The lower coercivity for the NRs in comparison with the NTs pointed towards a lower effective anisotropy for the former. In addition, the fact that the normalized remanence in both cases was much lower than the expected value for magnetically blocked and randomly aligned MNPs (M_r_/M_S_ ~ 0.5, according to the Stoner–Wohlfarth model [52]) confirmed the important role of the magnetic interactions in our samples. Concerning this, different studies have revealed that in aggregated nanoparticles, by increasing the size of the aggregate, the effect of the intraparticle interactions becomes increasingly relevant [25,53,54,55]. Therefore, as both NRs and NTs were formed by nanograins of a similar volume (~2600 nm^3^) but the total volume of the NTs was nearly 15 times greater than that of the NRs, the intraparticle interactions were going to be more relevant for the NTs than for the NRs. This has an important effect in their heating efficiency as we will see later. In this respect, we compared the obtained M-H loops with those measured for the 15 nm nanospheres [56] and 65 × 6 nm nanorods [35], which had a size similar to the nanograins of the NRs and NTs, respectively (see Figure S2 in the Supplementary Information). As depicted, the M-H loops for the nanospheres and nanorods presented a nearly null coercive field (<20 Oe) and a normalized remanence (<0.05), thereby showcasing the importance of the interactions when these nanospheres and nanorods formed part of the multigranular NRs and NTs, respectively. Curiously, in both cases, the M_S_ value of the NRs and NTs was lower than that of nanospheres and nanorods. This would support the presence of a higher magnetic disorder in these multigrain nanostructures in comparison with their nanograins, as mentioned before.

In order to get an estimation of the effective anisotropy in these samples, we carried out radio frequency (RF) transverse susceptibility (TS) measurements. The TS method is a precise tool for investigating the anisotropic magnetic properties of different magnetic systems including MNPs [57,58]. TS spectra typically display peaks at the anisotropy fields (H_K_) and switching fields (H_S_) as the DC field is swept from a positive to a negative saturation. The bipolar TS curves taken at 300 K for the NRs and NTs are depicted in Figure 3a. In both cases, two peaks could be observed indicating that the switching peaks were merged with the anisotropic peak. This resulted in a slight difference in the positive and negative H_K_ values together with a difference in the peak height. The estimated H_K_ values are represented as a function of the temperature in Figure 3b. The H_K_ values at room temperature were 450 and 510 Oe for the NRs and NTs, respectively. These values were appreciably higher than those obtained for the coercive field. An increase in H_K_ has been previously related to an increase in the strength of dipolar interactions [57]. Therefore, in our case, these results would suggest the presence of stronger intraparticle interactions for the NTs in comparison with the NRs. As shown in Figure 3b, with a decreasing temperature, these values remained more or less constant but they rapidly increased below the Verwey transition (~110 K), reaching 690 and 595 Oe below 30 K for the NTs and NRs, respectively. Therefore, even if the ZFC/FC M-T measurements of the Verwey transition were not evident, the TS measurements clearly revealed its presence. Finally, we must remark that below 30 K, the H_K_ value remained constant, probably due to the system entering into a frozen collective magnetic state [57,59].

In addition, another parameter that could be analyzed from the TS curves was the peak height difference, ɳ. It has been previously shown that the ɳ tends to increase with an increasing interaction strength [57]. In this respect, in Figure 3c, we plotted the ɳ(T) curves for both the NRs and NTs. In the whole range of temperatures analyzed, the peak height difference (ɳ) was lower for the NRs than for the NTs, supporting once again the presence of stronger intraparticle interactions for the NTs. The thermal evolution of ɳ followed a trend similar to that of H_K_ vs. T. The maxima in the ɳ(T) curves marked the Verwey transition (~110 K). In addition, we observed that in the range of temperatures studied, ɳ calculated from a positive and a negative saturation showed a difference, which could be due to the dynamic state of the system (Figure S3 in the Supplementary Information). The difference in ɳ from the positive and the negative saturation was higher in the NRs compared with the NTs. This was also observed in the difference between the ZFC and FC magnetization curves.

In Figure 3d, we represented the thermal evolution of the maximum change in TS (Δχ_T_/χ_T_)_max_ curves. This parameter, which was sensitive to changes in the dynamic state of the system, showed a similar behavior at a low temperature with a decrease below ~100 K ascribed to the Verwey transition of Fe_3_O_4_. However, we observed several differences at higher temperatures with a sudden increase in the (Δχ_T_/χ_T_)_max_ above 200 K for the NRs and 250 K for the NTs. This could be related to the thermal disorder overcoming the magnetic order with an increasing temperature [57]. The fact that this happened at lower temperatures for the NRs than for the NTs supported the weaker effective anisotropy of the NRs in comparison with the NTs.

Therefore, the magnetic measurements indicated that both NRs and NTs presented a similar magnetic behavior with the expected structurally-coupled magnetic transitions intrinsic to Fe_3_O_4_ but there were a few quantitative differences related to the higher effective anisotropy of the NTs, which could be attributed to the stronger role of intraparticle interactions in these samples. In order to ascertain how these differences affected their efficiency as magnetic hyperthermia mediators, we carried out AC magnetometry measurements in these samples and obtained their SAR vs. H curves. The AC magnetometry measurements allowed us to gain an insight into the differences of the hysteresis losses and, hence, the heating efficiency obtained for both samples. In addition, AC hysteresis loops can allow us to better understand the nature and origin of the magnetic interactions [25].

**Figure 4:**
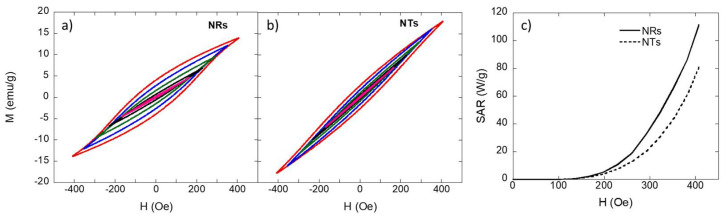
AC magnetometry loops obtained for (**a**) the iron oxide NRs and (**b**) NTs at 300 kHz with H_max_ = 400 Oe. (**c**) SAR vs. H curves derived from the AC hysteresis loop area (H = 0–400 Oe, f = 300 kHz).

We have represented the AC hysteresis loops for the NRs and NTs in Figure 4a,b. As depicted, in both cases, for fields up to 400 Oe we obtained minor loops with a maximum magnetization (≈15 emu/g), far from the M_S_ values reached in the DC magnetic measurements. The fact that even at 400 Oe we obtained narrow minor loops made sense considering the high anisotropy field values exhibited near room temperature by both NRs (H_K_ = 450 Oe) and NTs (H_K_ = 510 Oe). As has been described in the literature, we could distinguish two regimes in the heating efficiency of MNPs under AC magnetic fields: at low fields, H << H_K_, the power absorption is mainly caused by viscous losses in the system and this regime is characterized by small hysteresis loop areas [17,60]. On the other hand, at higher fields, H >> H_K_, hysteresis losses dominate and the area of the hysteresis loops appreciably increases, eventually tending to saturate. In our case, in the range of AC fields applied, we were mainly working in the first regime. There were, nevertheless, several differences in the shape of the AC hysteresis loops for both samples with those of the NTs being slightly narrower and less squared. This could be related to the effect of intraparticle interactions: in the NTs, intraparticle interactions played a more important role than in the NRs and this gave rise to stronger demagnetizing effects that made the AC hysteresis loops narrower, as has been reported before [17,25,61].

As depicted in Figure 4c, we plotted the SAR vs. H curves computed from the area of the AC hysteresis loops [62]. Below 200 Oe, both NRs and NTs presented a negligible heating efficiency but above 200 Oe, the SAR values tended to increase; more rapidly for the NRs than for the NTs, reaching a maximum value of ~110 and 80 W/g, respectively, at 400 Oe. The obtained SAR values were smaller than those reported for other similar hollow nanostructures, e.g., Dias et al. [41] reported SAR values up to 426 W/g for magnetite NRs measured at 200 Oe and 300 kHz. Nevertheless, as depicted, the SAR vs. field curves were still far from saturation and, therefore, the SAR values could keep rising by increasing the applied field but this could pose a risk for the safety of the patient [18]. The current clinical safety limits indicate that the product of the field amplitude × frequency ≤ 5 × 10^9^ Am^−1^s^−1^ [63]. This implies that for a frequency of 300 kHz, as in our case, the maximum applied field should not be higher than 208 Oe. In this range, the NRs still provided a better heating efficiency than the NTs although in both cases the SAR values were very small. Therefore, these results indicated that at low fields, H << H_K_, the MNPs with a lower effective anisotropy performed better as heating agents than those with a higher effective anisotropy [25].

The effect of the intraparticle interactions in the heating efficiency of NRs and NTs could be even better visualized if we compared the AC hysteresis loops and the SAR vs. H curves of the NRs and NTs with those measured for MNPs very similar to their corresponding nanograins: 15 nm nanospheres (see [56]) and 65 × 6 nm nanorods (see [35]), respectively. As depicted in Figure S4 (see Supplementary Information), the AC hysteresis loops for both nanorods and nanospheres were more squared and saturated than those obtained for the NRs and NTs. The differences were especially remarkable in the case of the nanorods. This could also be observed in the SAR vs. H curves: the SAR values measured for the nanorods and nanospheres were higher than those obtained for the NTs and NRs for all of the fields analyzed. The nanorods and nanospheres reached a maximum SAR value of 300 W/g and 140 W/g, respectively, at 400 Oe and 300 kHz. These values were 2.7 and 1.7 times higher than those obtained for the NTs and NRs, respectively. The higher difference between the SAR values of the NTs and nanorods indicated the higher impact of the intraparticle interactions in the NTs compared with the NRs.

## 4. Conclusions

In summary, we showed that intraparticle magnetic interactions can play a crucial role in the heating efficiency of multigranular nanostructures. When the nanograins bind together to form this kind of nanostructure, the strength of the intraparticle interactions increases with the increasing volume. As we have seen in the case of NRs and NTs, this gives rise to an increase in the effective anisotropy of the nanostructures in comparison with the isolated nanograins, which tends to make them become magnetically blocked at the range of temperatures relevant for magnetic hyperthermia. These blocked magnetic nanostructures present high anisotropy fields at room temperature (450 Oe for NRs and 510 Oe for NTs), which deter their heating efficiency. This was precisely observed in the AC hyperthermia measurements carried out. In the range of fields analyzed (0–400 Oe), both NRs and NTs gave rise to minor loop hysteresis losses and therefore lower SAR values (110 W/g for NRs and 80 W/g for NTs) especially when compared with the isolated nanograins, which reached SAR values up to three times higher.

These results indicate that in the range of field amplitudes and frequencies currently relevant for clinical hyperthermia, multigranular nanostructures with a lower volume and therefore weaker intraparticle interactions are preferred as heating mediators. Therefore, in our case, multigranular NRs, despite their lower effective anisotropy and saturation magnetization, present a better heating efficiency compared with the NTs.

## Figures and Tables

**Table 1:** Dimensions of the iron oxide NRs and NTs. The length corresponds to the “height” of the hollow cylinder and the difference between the inner and outer diameters gives the wall thickness.

	Length (nm)	Inner Diameter (nm)	Outer Diameter (nm)	Wall Thickness (nm)
**NRs**	55 ± 5	55 ± 5	110 ± 15	55 ± 5
**NTs**	470 ± 45	110 ± 20	170 ± 20	55 ± 5

## Data Availability

Not applicable.

## Supplementary Materials

The following are available online at https://www.mdpi.com/article/10.3390/nano11061380/s1, Figure S1: The size distribution histograms for (a–c) NRs and (d–f) NTs, Figure S2: TEM images and size distributions of 15 nm nanospheres (a,b) and 65×6 nm nanorods (d–f). In addition, the M-H loops measured at 300 K for the nanospheres (c) and the nanorods (g) are also included. The insets are a zoom-in of the low field region of these M-H loops., Figure S3: Peak height difference (*ɳ*) curves calculated from positive and negative saturation magnetization (*M*_sat_) for NRs and NTs., Figure S4: (a) AC loops and (b) SAR vs H curves for NTs, NRs, and 15 nm nanospheres and 65 × 6 nm nanorods, similar to the constituent nanograins.
