# Supplementary material for: Iron Oxide Nanorings and Nanotubes for Magnetic Hyperthermia: The Problem of Intraparticle Interactions
Source: Nanomaterials (Basel). 2021 May 24;11(6):1380. doi: 10.3390/nano11061380 (PMC8225017; doi:10.3390/nano11061380)
Supplement: Supplementary file 1 [file nanomaterials-11-01380-s001.zip › nanomaterials-1219934-supplementary.pdf]

SUPPLEMENTARY INFORMATION

Iron Oxide Nanorings and Nanotubes for Magnetic Hyperthermia: The Problem of Intraparticle Interactions

Raja Das [1,2,*], Javier Alonso Masa [3], Vijaysankar Kalappattil [4], Zohreh Nemati [4], Irati Rodrigo [5], Eneko Garaio [6], José Ángel García [7], Manh-Huong Phan [4] and Hariharan Srikanth [4,*]

1 Faculty of Materials Science and Engineering, Phenikaa University, Hanoi 12116, Vietnam
2 Phenikaa Research and Technology Institute (PRATI), A&A Green Phoenix Group, 167 Hoang Ngan, Hanoi 13313, Vietnam
3 Departamento CITIMAC, Universidad de Cantabria, 39005 Santander, Spain; alonsomasaj@unican.es
4 Department of Physics, University of South Florida (USF), Tampa, FL 33620, USA;
vijaysankar@mail.usf.edu (V.K.); zohre.nematy@gmail.com (Z.N.); phanm@usf.edu (M.-H.P.)
5 Departamento de Electricidad y Electrónica, Universidad del País Vasco (UPV/EHU), 48940 Leioa, Spain; irati.rodrigo@bcmaterials.net
6 Depto. de Física Aplicada, Universidad Pública de Navarra (UPN), 31006 Pamplona, Spain; eneko.garayo@unavarra.es
7 Depto. de Física, Universidad del País Vasco (UPV/EHU), 48940 Leioa, Spain; joseangel.garcia@ehu.eus
* Correspondence: raja@phenikaa-uni.edu.vn (R.D.); sharihar@usf.edu (H.S.)

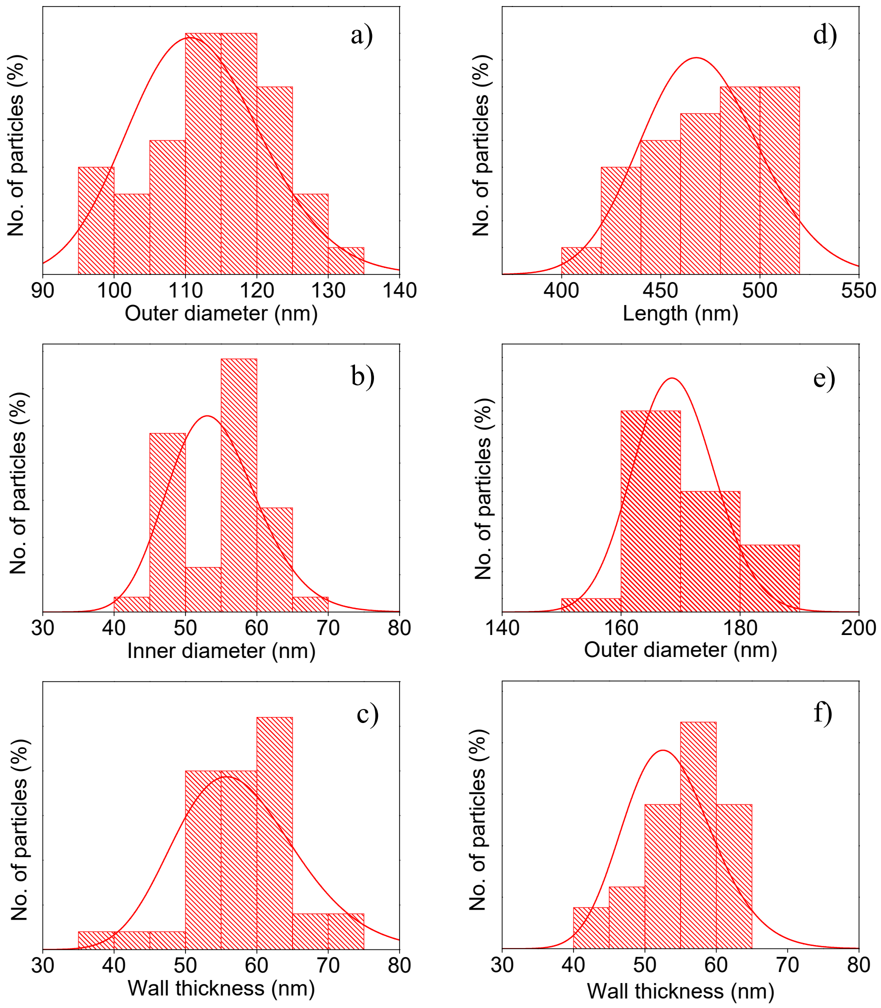

Figure S1. The size distribution histograms for (**a–c**) NRs and (**d–f**) NTs.

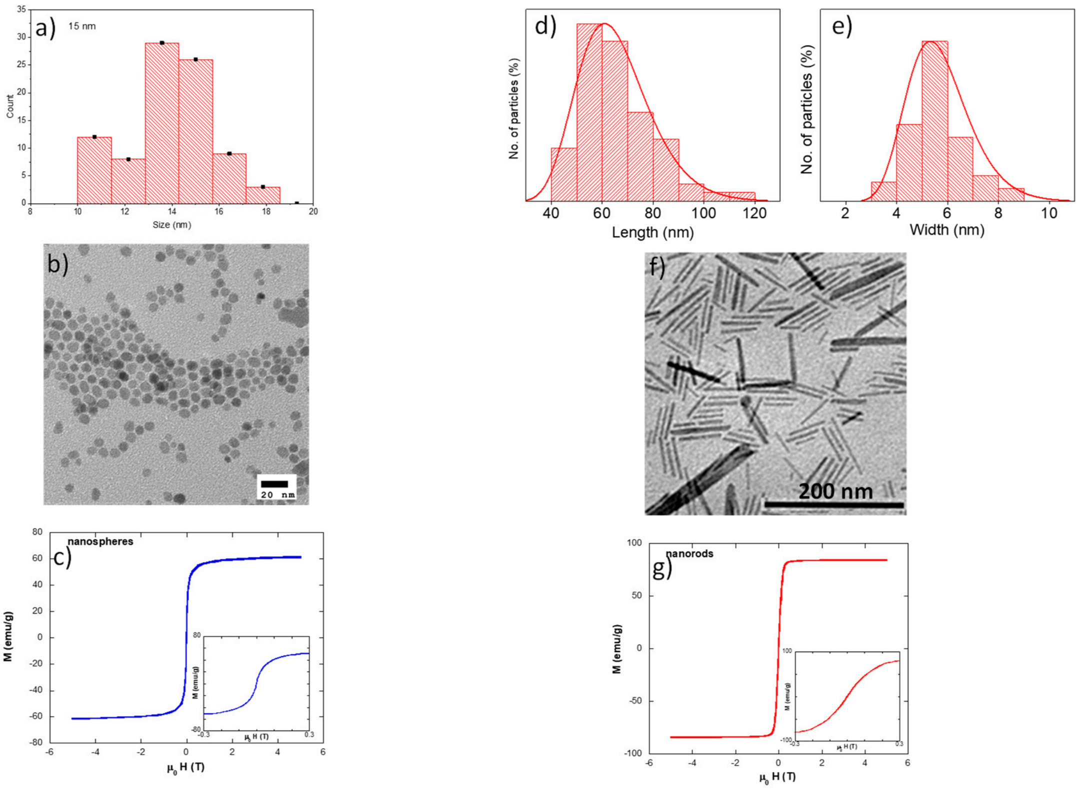

Figure S2. TEM images and size distributions of 15 nm nanospheres (**a**,**b**) and 65 × 6 nm nanorods (**d**–**f**). In addition, the M-H loops measured at 300 K for the nanospheres (**c**) and the nanorods (**g**) are also included. The insets are a zoom-in of the low field region of these M-H loops.

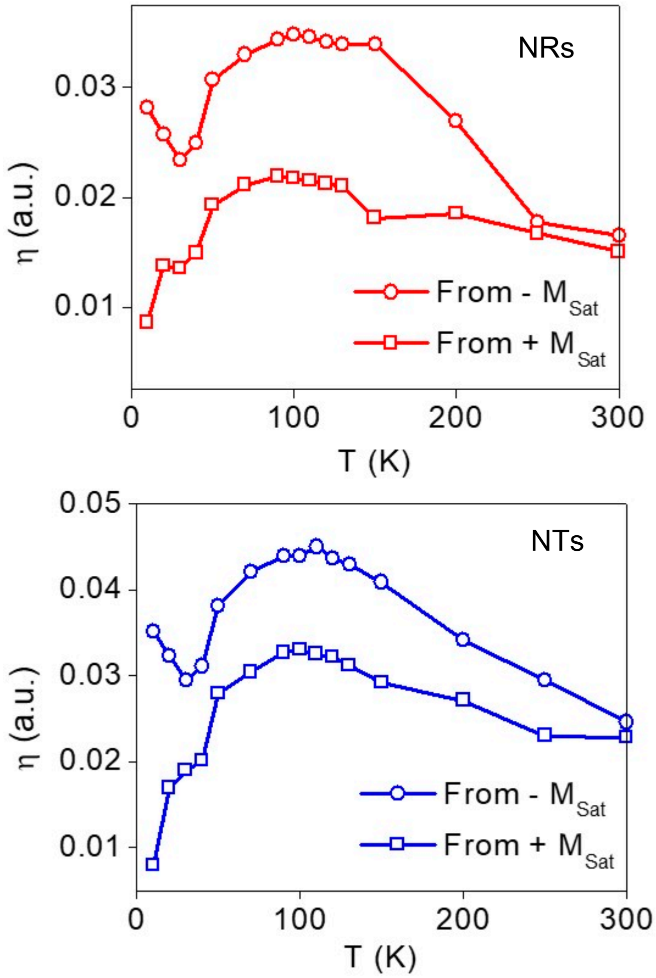

Figure S3. Peak height difference (*η*) curves calculated from positive and negative saturation magnetization (M_{sat}) for NRs and NTs.

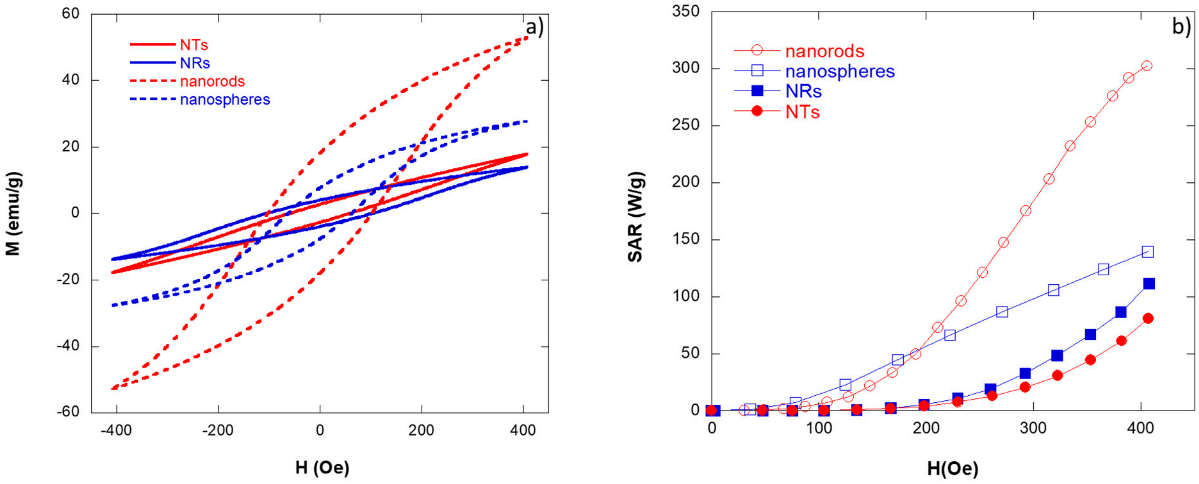

Figure S4. (**a**) AC loops and (**b**) SAR vs H curves for NTs, NRs, and 15 nm nanospheres and 65 × 6 nm nanorods, similar to the constituent nanograins.
